# Schizophrenia Polygenic Risk During Typical Development Reflects Multiscale Cortical Organization

**DOI:** 10.1016/j.bpsgos.2022.08.003

**Published:** 2022-08-24

**Authors:** Matthias Kirschner, Casey Paquola, Budhachandra S. Khundrakpam, Uku Vainik, Neha Bhutani, Benazir Hodzic-Santor, Foivos Georgiadis, Noor B. Al-Sharif, Bratislav Misic, Boris C. Bernhardt, Alan C. Evans, Alain Dagher

**Affiliations:** a Montreal Neurological Institute, McGill University, Montreal, Québec, Canada; b Department of Psychiatry, Psychotherapy and Psychosomatics, Psychiatric Hospital, University of Zürich, Zürich, Switzerland; c Division of Adult Psychiatry, Department of Psychiatry, University Hospitals of Geneva, Geneva, Switzerland; d Institute of Neuroscience and Medicine, Forschungszentrum Jülich, Jülich, Germany; e Institute of Psychology, Faculty of Social Sciences, Tartu, Estonia

**Keywords:** Cortical organization, Cortical thickness, Gene expression, Neurodevelopment, Polygenic risk, Schizophrenia

## Abstract

**Background:**

Schizophrenia is widely recognized as a neurodevelopmental disorder. Abnormal cortical development in otherwise typically developing children and adolescents may be revealed using polygenic risk scores for schizophrenia (PRS-SCZ).

**Methods:**

We assessed PRS-SCZ and cortical morphometry in typically developing children and adolescents (3–21 years, 46.8% female) using whole-genome genotyping and T1-weighted magnetic resonance imaging (*n* = 390) from the PING (Pediatric Imaging, Neurocognition, and Genetics) cohort. We contextualized the findings using 1) age-matched transcriptomics, 2) histologically defined cytoarchitectural types and functionally defined networks, and 3) case-control differences of schizophrenia and other major psychiatric disorders derived from meta-analytic data of 6 ENIGMA (Enhancing Neuro Imaging Genetics through Meta Analysis) working groups, including a total of 12,876 patients and 15,670 control participants.

**Results:**

Higher PRS-SCZ was associated with greater cortical thickness, which was most prominent in areas with heightened gene expression of dendrites and synapses. PRS-SCZ–related increases in vertexwise cortical thickness were mainly distributed in association cortical areas, particularly the ventral attention network, while relatively sparing koniocortical type cortex (i.e., primary sensory areas). The large-scale pattern of cortical thickness increases related to PRS-SCZ mirrored the pattern of cortical thinning in schizophrenia and mood-related psychiatric disorders derived from the ENIGMA consortium. Age group models illustrate a possible trajectory from PRS-SCZ–associated cortical thickness increases in early childhood toward thinning in late adolescence, with the latter resembling the adult brain phenotype of schizophrenia.

**Conclusions:**

Collectively, combining imaging genetics with multiscale mapping, our work provides novel insight into how genetic risk for schizophrenia affects the cortex early in life.

Schizophrenia is a multifaceted and heritable psychiatric disorder that is widely recognized to have a neurodevelopmental origin (1, 2, 3). Abnormal brain development likely predates the onset of clinical symptoms, which typically emerge in early adulthood (4). Genome-wide association studies (GWASs) support this hypothesis by showing that schizophrenia-related genes are involved in multiple neurodevelopmental processes (1,5). These genes may affect brain development, leading to vulnerability to environmental effects, and have been suggested to contribute to atypical cortical morphology, as previously observed in cohorts with a schizophrenia diagnosis (3).

Childhood and adolescent brain development involve dynamic and complex structural changes that are shaped by genetic and environmental factors (6, 7, 8, 9, 10). Longitudinal neuroimaging studies have consistently reported a global increase in cortical volume, thickness, and surface area that typically peaks in late childhood and is followed by decreases in adolescence (7,10,11). At the same time, regional maturational trajectories are heterochronous, whereby sensory areas mature earlier than transmodal cortex (10,12,13), shaping large-scale patterns of cortical differentiation (14).

The neurodevelopmental hypothesis of schizophrenia posits that cortical maturation is perturbed, producing widespread cortical abnormalities (15). Differences in cortical morphometry are consistently reported across different stages and clinical phenotypes of the schizophrenia spectrum (16, 17, 18). However, investigating neurodevelopmental features of schizophrenia requires a departure from classic case-control designs. Alternatively, focusing on genetic risk enables us to investigate neuroanatomical correlates in a large population-based cohort of children and adolescents without interacting with disease-related factors (e.g., medication and chronicity). Recent work shows an effect of polygenic risk scores for schizophrenia (PRS-SCZ) on cortical morphometry (19, 20, 21, 22), though not necessarily gray matter volume (23, 24, 25). Thus far, studies have focused almost exclusively on adult cohorts. Only one study has investigated adolescents (aged 12–21 years) and noted an association of PRS-SCZ with globally decreased cortical thickness among cannabis users (19). Discerning neurodevelopmental aspects of genetic risk for schizophrenia requires investigation of younger cohorts.

Understanding the relation of genetic risk for schizophrenia to neurodevelopment can be further enhanced by contextualizing imaging-derived phenotypes of polygenic risk with maps of cortical organization. At the cellular level, a range of processes associated with healthy cortical development, such as synaptic pruning, dendritic arborization, and intracortical myelination, are implicated in the development of schizophrenia and may produce regional cortical disruptions (26, 27, 28, 29). Recent advances in RNA sequencing (RNA-seq) of postmortem brain tissue (30) allow discernment of the relative contribution of cell types to patterns of atypical cortical morphometry (31,32). More complex interactions of microstructure and function on regional vulnerability may be captured by the groupings of cortical areas into cytoarchitectural types and functional networks. Indeed, recent studies of schizophrenia (17,33) and high PRS-SCZ in healthy adults (34) suggest differential sensitivity of histologically defined cytoarchitectural types (35) and functional networks (36). Finally, population-level effects of schizophrenia and other major psychiatric disorders can be used to illustrate the concordance of genetic risk for schizophrenia with disorder-related neuroanatomical phenotypes. Specifically, it can be tested how the association between genetic risk of schizophrenia and cortical morphometry in children and adolescents relates to shared and divergent neuroanatomical abnormalities across psychiatric disorders (32). Taken together, multiple scales of cortical organization can be used to provide a comprehensive description of the regional variations of an imaging-derived phenotype, such as genetic risk for schizophrenia.

Here, we address the relationship between PRS-SCZ and cortical organization in a large population-based cohort of typically developing children and adolescents (3–21 years) derived from the PING (Pediatric Imaging, Neurocognition and Genetics) study (37). We hypothesized that higher PRS-SCZ would be associated with atypical cortical morphometry (thickness, surface area, and volume). Then, we aimed to better understand the effect of PRS-SCZ on cortical morphometry by comparing the observed spatial patterns to cell type–specific gene expression, cytoarchitectural and functional differentiation, and cortical abnormalities seen in major psychiatric disorders. Finally, we examined age group–specific variations of high PRS-SCZ on cortical morphometry across different neurodevelopmental stages.

## Methods and Materials

### Subjects

Neuroimaging, demographic, and genetic data of typically developing children and adolescents were derived from the PING study (37). The PING dataset is a wide-ranging, publicly shared data resource comprising cross-sectional data from 1493 healthy subjects. Participants were recruited and evaluated in the greater metropolitan areas of Baltimore, Boston, Honolulu, Los Angeles, New Haven, New York, Sacramento, and San Diego using local postings and outreach activities. Written informed consent was given by parents for all PING subjects younger than 18 years. Participants between the ages of 7 and 17 years gave additional child assent, and all participants 18 years or older directly gave written informed consent. Exclusion criteria and more information about the PING cohort are described elsewhere (37). After quality control of genomic and imaging data (see below), a total of 390 participants from the PING dataset were included in this study. The mean age was 12.10 years (SD = 4.77, range = 3–21 years), and 46.8% were female (for more details, see Table S1).

### Genomic Data

Genomic data processing and calculation of PRSs followed a recent publication from Khundrakpam *et al.* (38). Specifically, 550,000 single nucleotide polymorphisms were genotyped from saliva samples using the Illumina Human660W-Quad BeadChip (Illumina, Inc.). Details on imputation and preprocessing can be found in Supplemental Methods. After single nucleotide polymorphism imputation and preprocessing, 4,673,732 variants were available for calculation of PRSs. Participants were filtered to have at least 0.95 loadings to the European genetic ancestry factor (coded as “GAF_europe” in the PING dataset), resulting in 526 participants. To capture and quantify population structure, the same participants were used to calculate the 10 principal components across the variants, excluding areas in high linkage disequilibrium with each other (--indep-pairwise 50 5 0.2) with PLINK 2.

The PRS-SCZ was trained using results from the latest GWAS on schizophrenia at the time of analysis (39). The GWAS was filtered for having imputation quality over 90. Polygenic scores were calculated with PRSice 2.30e (40). Clumping of the data was performed using PRSice default settings (clumping distance = 250 kb, threshold *r*^2^ = 0.1). To calculate the PRS-SCZ, we used the GWAS hits (*p* < 5 × 10^8^) cutoff criterion. This resulted in 86 variants common to the base and target datasets (Table S2). The choice of the GWAS hits threshold was made to increase the specificity of observed gene-brain associations for schizophrenia and to minimize the genetic overlap with other psychiatric disorders such as bipolar disorder (BD), which increases with lower PRS significance thresholds, including more single nucleotide polymorphisms. To illustrate this influence of PRS significance thresholds on the genetic overlap between schizophrenia and BD, we calculated PRS for BD by applying exactly the same processing pipeline (Supplemental Methods). In this sample, PRS-SCZ and PRS for BD did not correlate (*r* = 0.063) using the GWAS hits threshold chosen here, whereas applying lower significance thresholds, such as *p* = .05 and *p* = .1, resulted in moderate correlations (*r* = 0.254 and *r* = 0.286, respectively) (Figure S1).

### Image Acquisition and Preprocessing

Details on image acquisition and preprocessing are described elsewhere (37). The CIVET processing pipeline (http://www.bic.mni.mcgill.ca/ServicesSoftware/CIVET, page 2.1) (41) was used to compute cortical thickness, surface area, and cortical volume measurements at 81,924 regions covering the entire cortex, and quality control was performed by 2 independent reviewers (see Supplemental Methods for details). After quality control of the total 526 subjects who passed filtering for European genetic ancestry, a total sample size of 390 participants remained for all subsequent analyses.

### Statistical Analyses

#### Association Between PRS-SCZ and Cortical Morphometry

**Figure 1:**
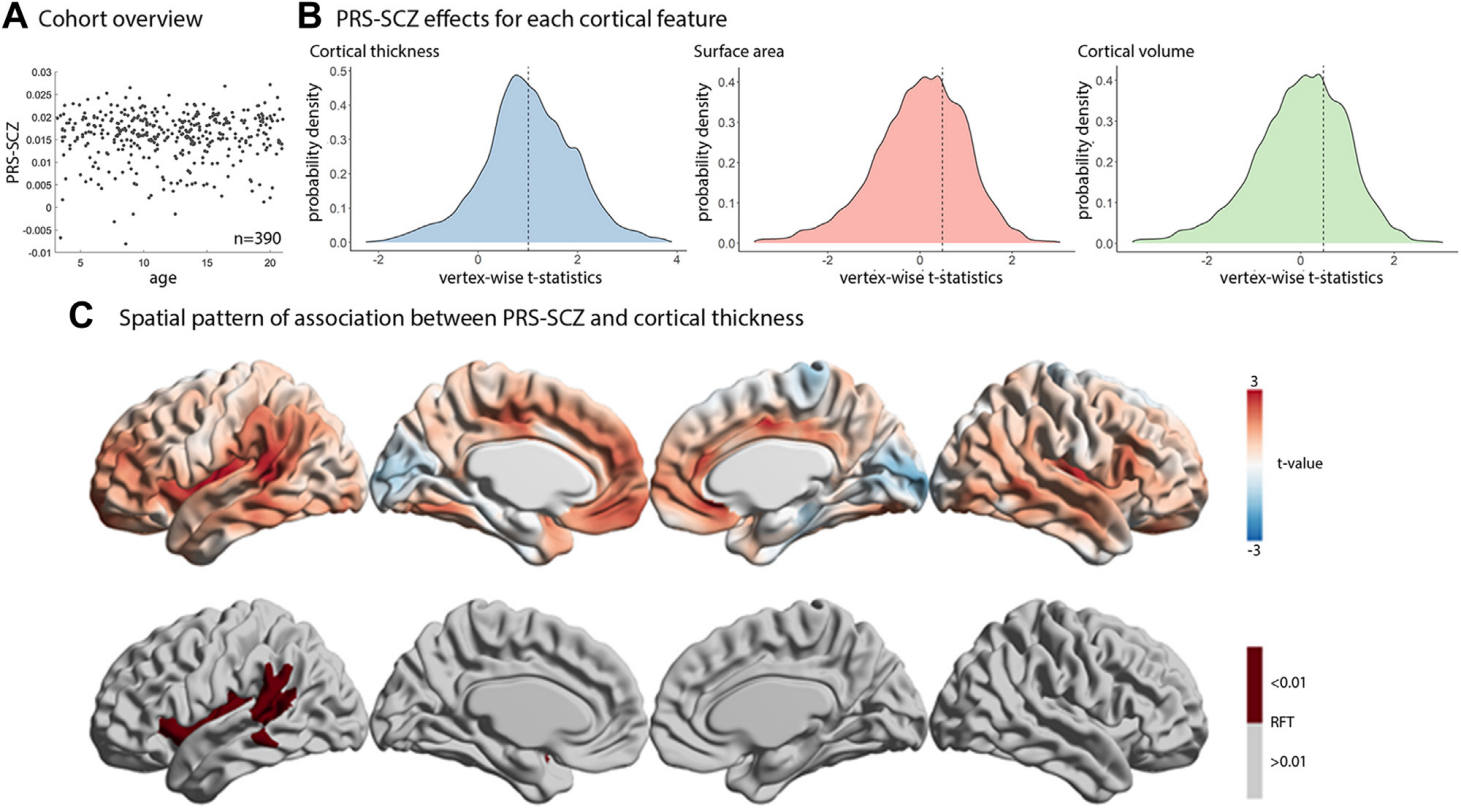
Association of polygenic risk scores for schizophrenia (PRS-SCZ) with cortical morphometry. **(A)** Scatterplot shows that PRS-SCZ varies across the age range, without being correlated with age. **(B)** Probability distributions show the variation in vertexwise *t* values of the association of PRS-SCZ with each cortical feature. Dashed lines represent the mean values of each cortical feature. Only cortical thickness was significantly shifted from 0. **(C)** Unthresholded (top) and thresholded (bottom) maps after random field theory (RFT) correction (*p* < .01) show the association of PRS-SCZ with cortical thickness. Unthresholded maps for surface area and cortical volume are Figures S2 and S3.

To identify the association of PRS-SCZ with vertexwise cortical thickness, surface area, and cortical volume, general linear models were applied using the SurfStat toolbox (http://www.math.mcgill.ca/keith/surfstat/) (42). Each cortical feature was modeled as

(1) $${\text{T}}_{\text{i}}=\text{intercept}\;+\;{\text{β}}_{1}\text{PRS-SCZ}\;+\;{\text{β}}_{2}\text{Age}\;+\;{\text{β}}_{3}{\text{Age}}^{2}+\;{\text{β}}_{4}\text{(PRS-SCZ∗Age)}\;+\;{\text{β}}_{5}{\text{(PRS-SCZ∗Age}}^{2}\text{)}+\;{\text{β}}_{6}\text{Sex}\;+\;{\text{β}}_{7}\text{PC10}\;+\;{\text{β}}_{8}\text{Scanner}\;+\;{\text{β}}_{9}\text{BrainVolume}\;+\;{\text{ε}}_{\text{i}}$$

where *i* is a vertex, PRS-SCZ is the PRS for schizophrenia, Age is years at the time of scan, PC10 are the first 10 principal components of genomic data to account for population stratification, *ε* is the residual error, and the intercept and the β terms are the fixed effects. Models with quadratic age terms were chosen because they fit the data better than models with only lower degree age terms (Supplemental Methods). For each cortical feature, vertexwise *t* statistics of the association with PRS-SCZ (β_1_PRS-SCZ) were mapped onto a standard cortical surface. To assess the significance of PRS-SCZ effects on each of the 3 different cortical features, whole-brain correction for multiple comparisons using random field theory (RFT) at cluster-level *p* ≤ .01 (43,44) was applied. Notably, only cortical thickness showed a significant association with PRS-SCZ after RFT correction (see Results, Figure 1); all subsequent analyses were thus restricted to cortical thickness only.

#### Cellular Composition of the Cortex and PRS-SCZ Effects on Cortical Thickness

We evaluated how the observed pattern of PRS-SCZ effects on cortical thickness relates to regional variations in the cellular compositions of the cortex. Given prior evidence (45) and histological validation (Supplemental Methods), we focused on components of the neuropil—namely glial cell processes, axons, dendritic trees, and neuron-to-neuron synapses—that are cortical tissues other than cell bodies or blood vessels. Neuropil-related gene expression was calculated by combining tissue-level RNA-seq (available online at http://development.psychencode.org/) (46) with gene lists of cell types, based on single-cell RNA-seq (30), and gene lists of neuron compartments, based on gene ontologies (47,48). Tissue-level RNA-seq provided expression levels of 60,155 genes in 11 neocortical areas (46). The areas were cytoarchitecturally defined in each specimen, supporting precise mapping and comparison across individuals. Crucially, we selected 12 brain specimens from the PsychEncode developmental dataset that were age-matched to the PING imaging cohort (3–21 years) because gene expression differs substantially between children and adults (49). Single-cell RNA-seq (30) provided specificity scores of each gene to glial cell types. For each type, we weighted the genes by the specificity score, then calculated the average across genes, across specimens, and within area. For each neuron compartment, we defined a list of marker genes using the Gene Ontology KnowledgeBase, then calculated the average expression of marker genes in each area and specimen. The annotated terms used were “neuron_to_neuron_synapse,” “dendritic_tree,” and “main_axon.” Next, we mapped the 11 areas to the cortical surface and extracted area-average PRS-SCZ effects on cortical thickness. The cortical areas were visually matched to the nearest parcel in a 200-parcel decomposition of the Desikan-Killiany, as performed in previous work (50). Finally, we tested the spatial similarity of cell type–specific gene expression with PRS-SCZ effects on cortical thickness using product-moment correlations. Statistical significance was determined relative to random reassignment permutation tests (10,000 repetitions).

#### Aggregation of PRS-SCZ Effects on Cortical Thickness by Cytoarchitectural Type and Functional Network

Given the hierarchical properties of cortical development (10) and given that disease-related imaging phenotypes are guided by different modes of cortical organization (17,33,51), we sought to contextualize the PRS-SCZ effects on cortical thickness by cytoarchitectural types and intrinsic functional networks. A whole-cortex atlas of cytoarchitectural types was acquired (https://github.com/caseypaquola/DMN) (52), which reflects an intersection of the classic Von Economo atlas of cortical areas (53,54) with a recent re-analysis of Von Economo micrographs that categorized the areas according to type (35). Cortical types synopsize degree of granularity, from high laminar elaboration in koniocortical areas, 6 identifiable layers in eulaminate III-I, poorly differentiated layers in dysgranular, and absent layers in agranular.

Functional networks were defined based on the Yeo atlas (https://github.com/ThomasYeoLab/CBIG) (36). The atlas reflects clustering of cortical vertices according to similarity in resting-state functional connectivity profiles acquired in 1000 healthy young adults. We assessed whether the PRS-SCZ effects on cortical thickness were stronger or weaker within each cortical class or functional network relative to spin permutations that preserve spatial autocorrelation (55,56) (see Supplemental Methods for details).

#### Pattern Similarity Between PRS-SCZ Effects and Cortical Abnormalities in Major Psychiatric Disorders

We assessed whether cortical thickness differences of PRS-SCZ relate to patterns of cortical thickness abnormalities observed in major psychiatric disorders including schizophrenia, BD, major depressive disorder (MDD), attention-deficit/hyperactivity disorder (ADHD), autism spectrum disorder, and obsessive-compulsive disorder. To this end, the PRS-SCZ–related *t*-statistic map was parcellated into 64 Desikan-Killiany atlas regions (57) and then correlated with the corresponding Cohen’s *d* maps derived from recently published meta-analyses by the ENIGMA schizophrenia (15), BD (58), MDD (59), ADHD (60), obsessive-compulsive disorder (61), and autism spectrum disorder (62) working groups, as implemented in the ENIGMA toolbox (63). Specifically, spatial pattern similarity of cortical Desikan-Killiany maps was examined using product-moment correlations. Statistical significance accounting for spatial autocorrelation was assessed with the spin permutation tests (10,000 repetitions) (55,56) implemented in the ENIGMA toolbox (63). The medial wall was assigned as a NaN and not included in any permuted correlation (64). Statistical significance was deemed where *p*_spin_ < .025 (two-tailed test), and false discovery rate (*p*_FDR_ < .05) was applied to control for multiple comparisons (*n* = 6).

#### Age Group Effects of PRS-SCZ on Cortical Thickness

We examined the distinctiveness of PRS-SCZ–related cortical thickness differences in different neurodevelopmental stages by dividing the sample into 3 age groups; early childhood (3–9 years, *n* = 145), early adolescence (10–15 years, *n* = 155), and late adolescence (16–21 years, *n* = 116) (65). The PRS-SCZ effect on cortical thickness was evaluated within each group using the above-mentioned general linear models (equation 1). However, the age term was centered to the mean of the group to focus on the effect within the specified developmental stage (8). Note that there was no correlation between age and PRS-SCZ scores (*r* = 0.058) (Figure 1A). We specifically examined whether the association of the PRS-SCZ effect with the adult cortical thickness phenotype of SCZ changed across age groups. To do so, we compared the product-moment correlation coefficients between age group and whole cohort *t*-statistic maps (66).

## Results

### Polygenic Risk for SCZ Is Associated With Greater Cortical Thickness

To test the association between PRS-SCZ and cortical morphometry in typically developing children and adolescents, we used T1-weighted MRI and whole-genome genotyping (*n* = 390) from the PING cohort (3–21 years, mean ± SD = 12.1 ± 4.7 years, 46% female) (Table S2). Vertexwise general linear models related cortical thickness with PRS-SCZ, controlling for age, sex, the first 10 principal components of genetic variants (to account for population stratification), scanner, and total brain volume. We found that higher PRS-SCZ was significantly associated with greater cortical thickness (RFT corrected, *p* < .01) but not surface area or cortical volume (Figure 1B; Figures S2 and S3). Overall, the unthresholded *t*-statistic map revealed that higher PRS-SCZ was associated with widespread increases in cortical thickness in association cortex but reduced cortical thickness in sensory areas (Figure 1C, top). Higher PRS-SCZ was associated with significantly thicker cortex in the left insula, left superior temporal gyrus, and left inferior parietal lobule (Figure 1C, bottom) (RFT corrected, *p* < .01). These results suggest a significant effect of PRS-SCZ on cortical thickness but not surface area or cortical volume in typically developing children and adolescents. As such, subsequent analyses are restricted to cortical thickness.

In the next step, we sought to examine how the PRS-SCZ–related cortical thickness increase in typically developing children and adolescents relates to different levels of cortical organization, including 1) cell type–specific gene expression, 2) cytoarchitectural and functional systems, and 3) cortical pattern of case-control differences from schizophrenia and other major psychiatric disorders.

### Alignment With Cell Type–Specific Gene Expression

**Figure 2:**
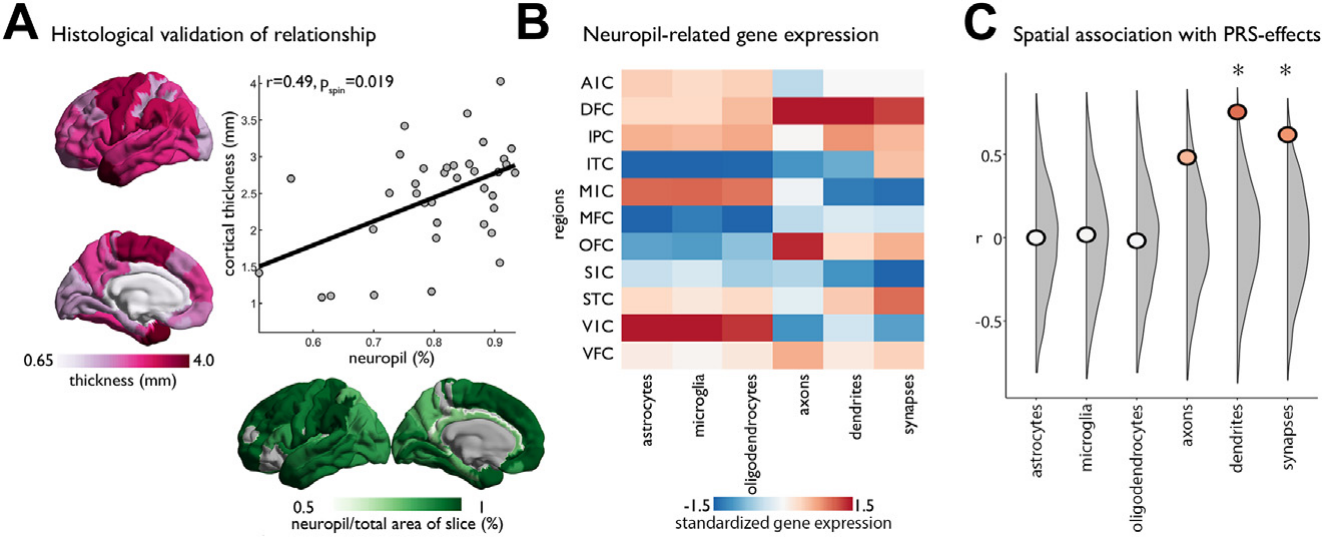
Decoding spatial patterns of polygenic risk scores for schizophrenia (PRS-SCZ) on cortical thickness. **(A)** Correlation of histological measurements of cortical thickness and neuropil from Von Economo and Koskinas (53). Cortical thickness is shown in pink and on the y-axis. Neuropil is shown in green and on the x-axis. **(B)** Gene expression varies across glial and neuron-related compartments of neuropil in 11 cortical regions. **(C)** Correlation of neuropil-related gene expression with the PRS-SCZ effects showed significant association with dendrites and synapses, compared with null distributions from permutation testing (gray). A1C, primary auditory cortex; DFC, dorsal frontal cortex; IPC, inferior parietal cortex; ITC, inferior temporal cortex; M1C, primary motor cortex; MFC, medial frontal cortex; OFC, orbital frontal cortex; S1C, primary somatosensory cortex; STC, superior temporal cortex; V1C, primary visual cortex; VFC, ventral frontal cortex.

Histological examinations have reported a null or minimal relationship between cortical thickness and neuron number in healthy brain samples (67, 68, 69). Instead, regional variations in cortical thickness show a strong association with neuropil (69), the portion of cortical tissue that excludes cell bodies or blood vessels (70). We examined whether cortical thickness differences related to PRS-SCZ mirrored regional variations in the neuropil composition of the cortex to generate hypotheses on the neuropil components affected by PRS-SCZ. To this end, we first validated the relation between cortical thickness and neuropil using tabular data and photomicrographs of Nissl stains (*r* = 0.49, *p*_spin_ = .018) (Figure 2A) (53,71). In contrast, neuronal density was not correlated with histologically defined cortical thickness (*r* = 0.08, *p*_spin_ = .678), which aligns with the findings of previous work (68,69). Then, we estimated regional variations in neuropil-related gene expression based on 6 cellular components (astrocytes, microglia, oligodendrocytes, axons, dendritic trees, and neuron-to-neuron synapses) by combining tissue-level RNA-seq (46) with single-cell RNA-seq for cell types (30) and gene ontologies for neuron compartments (47,48) (Figure 2B). Correlating PRS-SCZ effects on cortical thickness with neuropil-related gene expression, we found that PRS-SCZ effects are significantly associated with gene expression for dendritic trees (*r* = 0.755, *p*_perm_ = .006), synapses (*r* = 0.618, *p*_perm_ = .005), and at a trend level with axons (*r* = 0.481, *p*_perm_ = .069) (Figure 2C). In contrast, no significant correlation was observed with gene expression related to glial components of neuropil (Figure 2C). Together, these analyses suggest that greater cortical thickness with higher PRS-SCZ is observed in areas with greater dendritic and synaptic density.

### Contextualization by Cytoarchitectural Types and Functional Networks

**Figure 3:**
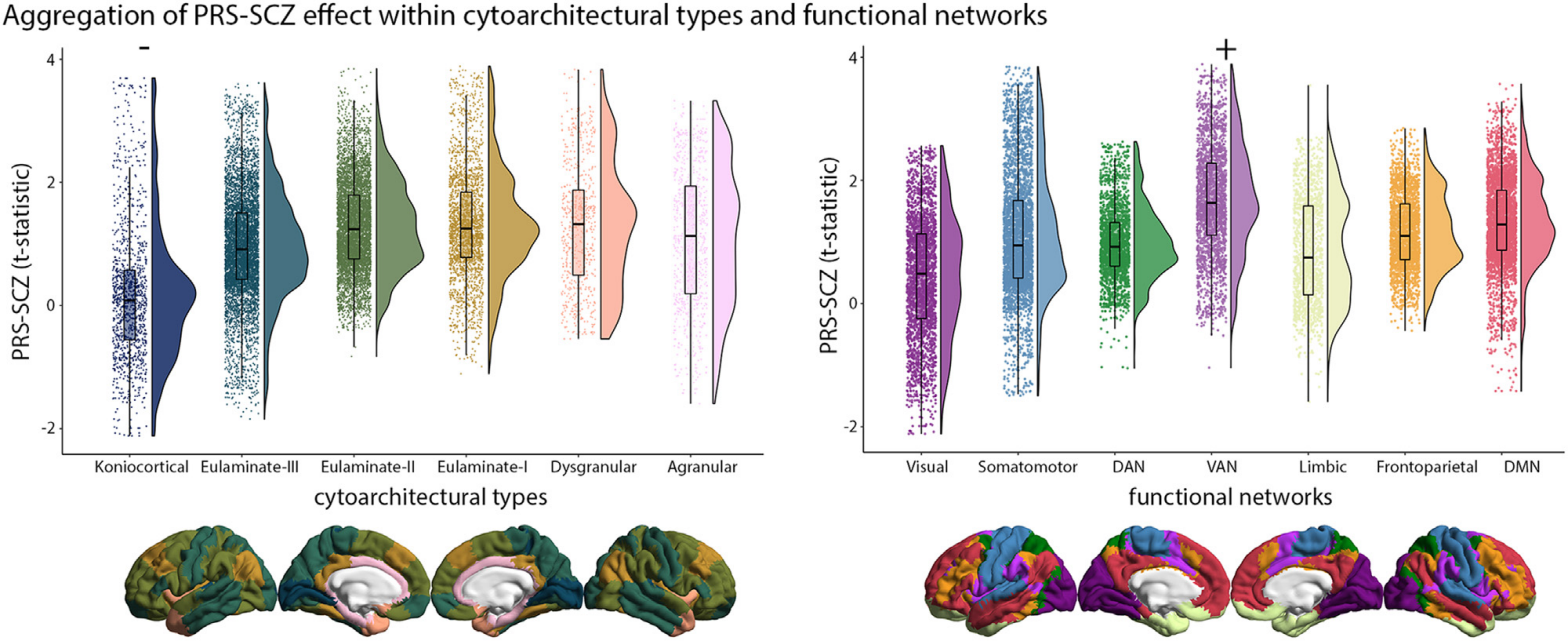
Aggregation of the polygenic risk scores for schizophrenia (PRS-SCZ) effect within cytoarchitectural types and functional networks. Raincloud plots show the distribution of the PRS-SCZ effect on cortical thickness stratified by cytoarchitectural type (35) and functional network (36). Relative to spin permutation null models, the koniocortical cortical type encompassed significantly lower *t* statistics, whereas the ventral attention network (VAN) encompassed significantly higher *t* statistics. DAN, dorsal attention network; DMN, default mode network.

Next, we aimed to determine whether the PRS-SCZ is preferentially associated with certain cytoarchitectural types or functional networks. Based on established atlases of cytoarchitectural and functional differentiation (35,36), we found that the PRS-SCZ effect was stronger than null models in the ventral attention network (median ± SD = 1.64 ± 0.88, *p*_spin_ = .004). Conversely, the PRS-SCZ effect was weaker or more negative than null models in the koniocortical type (i.e., primary sensory areas) (median ± SD = 0.08 ± 1.10, *p*_spin_ = .008) (Figure 3).

### Thickness Signatures of PRS-SCZ and Major Psychiatric Disorders

**Figure 4:**
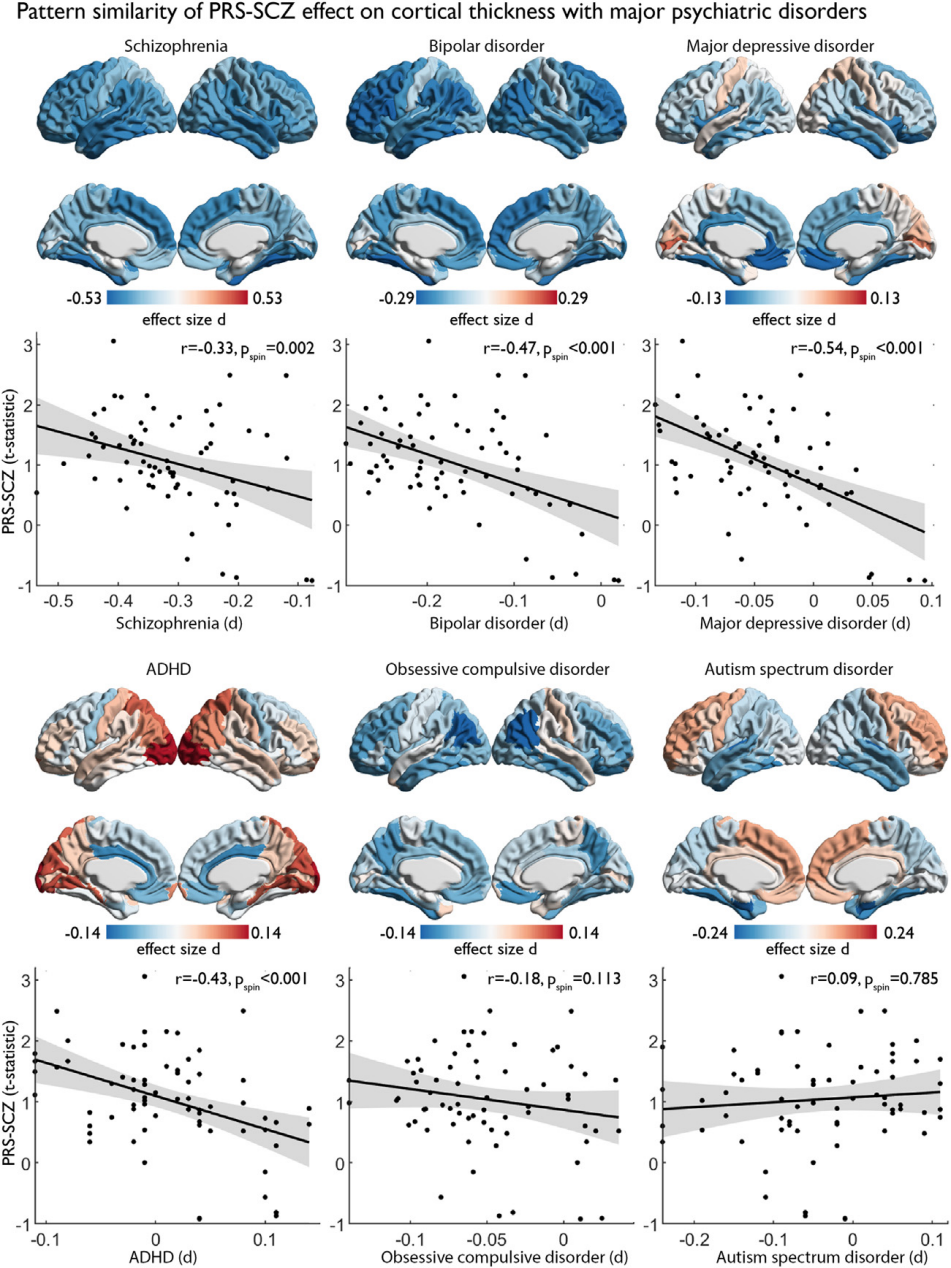
Pattern similarity of polygenic risk scores for schizophrenia (PRS-SCZ) on cortical thickness with major psychiatric disorders. Cortical surfaces show the effect size of schizophrenia, bipolar disorder, major depressive disorder, attention-deficit/hyperactivity disorder (ADHD), obsessive-compulsive disorder, and autism spectrum disorder diagnosis on cortical thickness from ENIGMA (Enhancing Neuro Imaging Genetics through Meta Analysis) meta-analyses of each disorder (63). Statistical significance was deemed where *p*_spin_ < .025 (two-tailed test) and false discovery rate (FDR) (*p*_FDR_ < .05) was applied to control for multiple comparisons (*n* = 6). Scatterplots show the correlation of each map with PRS-SCZ effect on cortical thickness.

We assessed whether PRS-SCZ effects on cortical thickness relate to abnormal cortical thickness patterns observed in case-control meta-analyses (Cohen’s *d* maps) of schizophrenia and other psychiatric illnesses. The PRS-SCZ–related cortical thickness increase showed a negative correlation with schizophrenia-related cortical abnormalities (*r* = −0.326, *p*_spin_ = .0022). In addition, we found similar negative correlations to cortical abnormalities in BD (*r* = −0.466, *p*_spin_ < .001), MDD (*r* = −0.538, *p*_spin_ < .001), and ADHD (*r* = −0.430, *p*_spin_ < .001) but not obsessive-compulsive disorder or autism spectrum disorder (Figure 4). To further test whether the correlation between PRS-SCZ–related cortical thickness differences and schizophrenia-related cortical thinning was significantly different compared with the correlations observed in non-SCZ psychopathologies, we applied pairwise comparisons of the correlation coefficients of schizophrenia with those from BD, MDD, and ADHD using the R package cocor (72) and the confidence interval test from Zou (73). The confidence intervals for each comparison of correlations *r* included zero (correlation *r*: SCZ vs. BD, 95% CI, −0.01 to 0.30; SCZ vs. MDD, 95% CI, −0.03 to 0.45; SCZ vs. ADHD, 95% CI, −0.20 to 0.39). This shows that the correlation of PRS-SCZ cortical thickness differences with the schizophrenia-related cortical thickness abnormalities was not significantly different compared with the correlations with each of the other major psychiatric disorders. Altogether, cortical regions showing PRS-SCZ–related greater thickness are those with the strongest thinning across disease maps of schizophrenia and genetically related affective disorders (e.g., BD, MDD, and ADHD).

### Age Group Effects of PRS-SCZ

**Figure 5:**
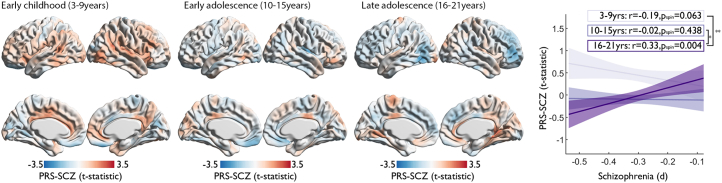
Age group–based polygenic risk scores for schizophrenia (PRS-SCZ) effect on cortical thickness. Cortical surfaces show unthresholded maps of PRS-SCZ effect on cortical thickness within age groups. Line plot shows how the relationship (linear regression with 95% confidence intervals) of PRS-SCZ with the SCZ-related pattern of abnormalities (Figure 4) changes from negative to positive across the age groups. Significant difference in correlation coefficients: ∗*p* < .05 and ∗∗*p* < .01.

Schizophrenia-related genes are implicated in neurodevelopmental processes, and as such, the effect of PRS-SCZ on cortical thickness likely varies with age. Although the cross-sectional nature of this cohort prohibits mapping the individual trajectories of cortical development, we sought to approximate developmental variation in the effect of PRS-SCZ by estimating age group effects of early childhood (3–9 years), early adolescence (10–15 years), and late adolescence (16–21 years) in the cohort. Because of the smaller sample sizes within each age group, effects were in general smaller compared with the main effect and did not survive multiple comparison corrections, with the exception of a small cluster in the right rostral anterior cingulate cortex for the early childhood group. Higher PRS-SCZ was associated with greater cortical thickness in early childhood. However, the pattern differed in the older age groups (Figure 5). We detected a significant difference in the correlation coefficients between early childhood and late adolescence (*z* = 2.84, *p* = .002), as well as early adolescence and late adolescence (*z* = 1.84, *p* = .033). Furthermore, PRS-SCZ–related cortical thickness increases in early childhood correlated negatively with schizophrenia-related cortical abnormalities, whereas PRS-SCZ–related cortical thinning in late adolescents correlated positively (Figure 5). To further inspect the age-related change in the PRS-SCZ effect on cortical thickness, we repeated the analysis using the entire cohort and iteratively shifting the age centering from 3 to 21 in 1-year intervals. Higher PRS-SCZ was associated with greater cortical thickness in the 3- to 6-year age-centered models (Figure S4), closely resembling the results from the main analysis (Figure 1C), and survived multiple comparison corrections (Figure S4 bottom) (RFT corrected, *p* < .01). Finally, the observed nonlinear relation of PRS-SCZ on cortical thickness across different age groups was confirmed when examining the interaction effect of PRS-SCZ and Age^2^ on cortical thickness across the entire age range 3–21 years (Figure S5).

## Discussion

Combining imaging genetics with multiscale mapping, we characterized the effect of PRS-SCZ on cortical morphometry across different scales of cortical organization. We found that higher PRS-SCZ was associated with greater cortical thickness in typically developing children and adolescents, while surface area and cortical volume showed only subtle associations with PRS-SCZ. We further provided evidence that PRS-SCZ preferentially affects areas with heightened expression of dendrites and synapses and that the PRS-SCZ–related cortical differences accumulate in cytoarchitecturally and functionally defined cortical systems. We also found that the PRS-SCZ–related cortical pattern mirrors cortical thinning related to schizophrenia and other major psychiatric disorders. Finally, age group models suggested a potential trajectory from PRS-SCZ–associated cortical thickness increase in early childhood toward thinning in late adolescence, spatially resembling the adult brain phenotype of schizophrenia.

Our cell type–specific gene expression approach enabled cross-modal exploration of the relation of genetic risk for schizophrenia with expression levels of neurons and glia. Our findings support and extend upon postmortem analyses, which demonstrate abnormal dendritic and synaptic density in individuals with schizophrenia [see (74) for a recent meta-analysis]. Another recent study suggested that differences in cortical thickness across multiple psychiatric disorders (including schizophrenia) are associated with pyramidal cell gene expression, a gene set enriched for biological processes of dendrites (e.g., dendritic arborization and branching) (75), as well as synaptic function (32). The present findings extend this work by demonstrating a relation between gene expression of dendrites and synapses with PRS-SCZ–related cortical differences during neurodevelopment.

System-specific contextualization revealed that PRS-SCZ effects on cortical thickness were spatially distributed, with the ventral attention network being preferentially sensitive to PRS-SCZ, while koniocortical type cortex was mostly spared from its influence. This cortical thickness pattern of PRS-SCZ closely mirrors recent observations in patients with schizophrenia showing stronger brain abnormalities in the ventral attention network, while primary cortex, as defined by von Economo, was relatively spared (33). Altogether, these findings demonstrate that system-specific differentiations of PRS-SCZ–related cortical thickness differences during neurodevelopment reflect cortical abnormalities of schizophrenia, suggesting some neuroanatomical continuity between polygenic risk and clinical phenotype.

Longitudinal data and case-control meta-analysis have shown that the development of psychosis in high-risk adolescents is associated with progressive loss of cortical thickness in several areas of the association cortex (18,76). Of note, the areas implicated in these studies overlap considerably with those showing increased cortical thickness in early childhood and more pronounced cortical thinning in adolescents with higher PRS-SCZ in this study. We further observed that the pattern of PRS-SCZ–related cortical thickening was associated with areas of cortical thinning in schizophrenia, BD, MDD, and ADHD. This cross-disorder overlap notably mirrors the genetic and phenotypic correlation between these disorders (32,77) and is in line with recent work in neurodevelopmental cohorts reporting a transdiagnostic mode of genome-connectome covariation (78) and shared features of frontotemporal dysconnectivity of general psychopathology (79). Collectively, these findings support the relevance of transdiagnostic gene-brain and brain-psychopathology phenotypes during neurodevelopment. While we do not know the cause of increased cortical thickness in our sample, converging evidence supports the idea that reduced cortical thickness in adults with schizophrenia results from loss of neuropil, and specifically synapses. For example, postmortem studies in schizophrenia demonstrate synaptic loss (74); many genes implicated in schizophrenia are associated with synapses or synaptic pruning (5,80,81); and regional variations in cortical thickness correlate with neuropil (69). It is conceivable that the PRS-SCZ is associated with delayed pruning and an excess of synapses for age, which in turn may render the affected brain regions vulnerable to catastrophic synaptic loss during the emergence of psychosis.

The association of PRS-SCZ with greater cortical thickness in early childhood raises the question of how the genetic risk of schizophrenia contributes to abnormal developmental trajectories. Given that the transmodal areas identified in this analysis, such as the insula, exhibit modest cortical thinning from 3 to 21 years (82, 83, 84), our results align with an amplified trajectory (i.e., higher peak, steeper decline) and/or delayed cortical thinning in early childhood. Related to the complexity and heterochronicity of cortical maturation during childhood and adolescents (82), polygenic disorders can involve multiple types of abnormal trajectories, occurring simultaneously or sequentially (85,86). Amplified or delayed trajectory of transmodal area morphometry may represent a core motif of cortical development in children with high PRS-SCZ. The PRS-SCZ includes multiple genetic factors, however, and their individual variation may produce heterogeneity in cortical development within high PRS-SCZ individuals.

The lack of associations between PRS-SCZ and surface area is in line with previous observations in adults (22) and meta-analytic evidence showing weaker surface area abnormalities compared to cortical thickness in schizophrenia (15). The divergent effects of PRS-SCZ on cortical thickness and surface area observed in this study might further be explained by different underlying genetic architecture of both cortical features. The mechanisms underlying cortical thickness versus surface area are often placed in the context of Rakic’s radial unit hypothesis, which proposes that surface area reflects the number of cortical columns, while thickness reflects the volume of each column (87). Using bioinformatic analyses, Grasby *et al.* (88) revealed that cortical thickness is influenced by genetic variants reflecting processes during mid fetal development including, myelination, branching, and pruning, while total surface area has been related to altered gene regulation in neural progenitor cells during fetal development. Collectively, these findings suggest that schizophrenia-related genetic variations exert a greater influence on neurodevelopmental processes altering cortical thickness than surface area.

This study should be interpreted in light of the cross-sectional nature of the dataset, which limits the ability to map individual longitudinal trajectories. The convergence of the observed findings with gene expression and neuroanatomical studies of schizophrenia support multiscale continuity between polygenic risk and clinical phenotype of schizophrenia. However, given the low familial risk and the relative absence of other biological or environmental risk factors for schizophrenia in the study cohort, interaction between PRS-SCZ and other biological and environmental risk factors could not directly be assessed and warrants further investigation. In addition, although mapping polygenic risk profiles on neuroimaging-derived phenotypes is a useful approach to further our understanding of genetic influence on neuroanatomical signatures related to schizophrenia risk, this method is limited by the fact that it does not allow direct translation into underlying biological mechanisms. Future research could therefore be enhanced by larger datasets with longitudinal designs and longer follow-up to determine which individuals will develop psychosis or other mental disorders. Finally, the observed PRS-SCZ–related cortical thickness increase in early childhood (age 3–9) highlights the need for large-scale initiatives targeting this age range.

### Conclusions

This study provides novel evidence on the cellular basis and developmental trajectory of cortical thickness differences related to genetic risk for schizophrenia that may help to refine the neurodevelopmental hypothesis of schizophrenia. More generally, this work illustrates how maps of cortical organization can enrich descriptions of imaging-derived phenotypes related to genetic risk for mental illnesses. Altogether, this integrative framework combining imaging genetics and multiscale mapping could advance our understanding of the complex associations between individual genetic profiles and cortical organization across multiple psychiatric and neurologic conditions.

## Supplementary Material

Supplementary Data

Key Resources Table
