# Supplementary material for: Schizophrenia Polygenic Risk During Typical Development Reflects Multiscale Cortical Organization
Source: Biol Psychiatry Glob Open Sci. 2022 Aug 24;3(4):1083–93. doi: 10.1016/j.bpsgos.2022.08.003 (PMC10593879; doi:10.1016/j.bpsgos.2022.08.003)
Supplement: Supplementary Data [file mmc1.pdf]

SUPPLEMENTARY INFORMATION

Schizophrenia Polygenic Risk During Typical Development Reflects Multiscale Cortical Organization

Table S1. Sample Characteristics

Variables	Sample (n=390)
Age, mean (SD)	12.10 (4.77)
Gender (%)	46.80 % female
Handedness (n=390)	left=38; right=338; mixed=14
Socioeconomic Status, mean (SD)	11.76 (1.89)
Family History (%)	
Schizophrenia family risk	0.056
Bipolar family risk	0.171
Depression&Anxiety family risk	0.529
ADHD family risk	0.256

Image acquisition and pre-processing

Details on image acquisition and pre-pre-processing are described elsewhere (1). Each site administered a standardized structural MRI protocol including a 3D T1-weighted inversion prepared RF-spoiled gradient echo scan using prospective motion correction (PROMO), for cortical and subcortical segmentation. The CIVET processing pipeline, (http://www.bic.mni.mcgill.ca/ServicesSoftware/CIVET) involved the following steps: T1-weighted image were first non-uniformity corrected, and then linearly registered to the Talairach-like MNI152 template (established with the ICBM152 dataset). Using the template mask, the non-uniformity correction was repeated. In a next step, the non-linear registration from the resulting volume to the MNI152 template was computed priors were obtained from the transform to segment the image into GM, WM, and cerebrospinal fluid. Inner and outer GM surfaces were then extracted

using the Constrained Laplacian-based Automated Segmentation with Proximities (CLASP) algorithm, and cortical thickness was estimated in native space using the linked distance between the two surfaces at 81,924 vertices. Each subject's cortical thickness map was blurred using a 30-millimeter full width at half maximum surface-based diffusion smoothing kernel to impose a normal distribution on the cardiometry data, and to increase the signal to noise ratio. Quality control (QC) of these data was performed by two independent reviewers. Data with motion artifacts, a low signal to noise ratio (lower than 800), artifacts due to hyperintensities from blood vessels, surface-surface intersections, or poor placement of the grey or white matter (GM and WM) surface for any reason were excluded of the total 526 subjects that passed filtering for European genetic ancestry. Next, filtering for individuals with information for demographics (age, sex, ethnicity and scanner) and those which passed quality control, resulted in a final sample of 390 participants.

Genomic data imputation and preprocessing

Genomic data processing and calculation of polygenic risk scores followed a recent publication from Khundrakpam and colleagues (2). Specifically, 550,000 single nucleotide polymorphisms (SNPs) were genotyped from saliva samples using the Illumina Human660W-Quad BeadChip.

Data were prepared for imputation using the "imputePrepSanger" pipeline (https://hub.docker.com/r/eauforest/imputeprepsanger/), implemented on CBRAIN (Sherif et al., 2014) and the Human660W-Quad_v1_A-b37-strand chip as reference. Starting from Plink genotype files, we adjusted the strand, the positions, the reference alleles to match the HRC panel and performed quality control steps, resulting in a final vcf file. Specifically, using Plink, we excluded SNPs and subjects with more than 10% of missing data (--mind 0.1, --geno 0.1) and kept only common SNPs (--maf 0.05) passing the Hardy-Weinberg equilibrium test (-- hwe 5e-8). Indels, palindromic SNPs, SNPs with differing alleles, SNPs with no match to the reference panel, SNPs with > 0.2 allele frequency difference to the reference, and duplicates were also removed,according to the pipeline. The Sanger Imputation Service (3) was used for data imputation, applying default settings and the Haplotype Reference Consortium, HRC (http://www.haplotype-reference-consortium.org/) as the reference panel. The imputed SNPs were further filtered with Plink v1.90b6.17 and v2.00a3LM (4). All imputed SNPs had INFO scores R^2>0.9 and were filtered again using a more stringent filtration criteria (–mind 0.05, --geno 0.05, --maf 0.05, --hwe 1e6, --maf 0.05, --max-alleles 2) and heterozygosity rates (--het) 3 standard

deviation (SD) units from the mean. We further omitted data from MHC complex (chr6 28477797 33448354 , https://www.ncbi.nlm.nih.gov/grc/human/regions/MHC?asm=GRCh37).

After data imputation and preprocessing, 4,673,732 variants were available for calculation of polygenic scores. Participants were filtered to have at least 0.95 loadings on the European genetic ancestry factor (coded as "GAF_europe" in the PING dataset), resulting in 526 participants. To model population structure, the same participants were used to calculate the top 10 principal components across the variants, excluding areas in high LD with each other (--indep-pairwise 50 5 0.2) with Plink 2.

The PRS-SCZ was trained using results from latest GWAS on schizophrenia at the time of analysis (5). The GWAS was filtered for having imputation quality over 90. Polygenic scores were calculated with PRSice 2.30e (6). The data were clumped as per PRSice default settings (clumping distance = 250kb, threshold r2 = 0.1), using the GWAS hits ($p < 5x10^{*-08}$) cut-off criterion. After matching our genotyped dataset post-imputation with the GWAS hits, the PRS-SCZ was based on 86 variants.

The PRS for bipolar disorder (PRS-BIP) was generated following exactly the same steps and using results from the latest GWAS on bipolar disorder at the time of analysis (5). After clumping the data as per PRSice default settings (clumping distance = 250kb, threshold r2 = 0.1), using the GWAS hits ($p < 5x10^{*-08}$) cut-off criterion and matching with available variants in the data, the PRS-BIP was based on 10 variants.

Table S2. List of 86 SNP included in PRS SCZ using GWAS hits ($p < 5x10^{*-08}$) cut-off criterion

CHR	SNP	BP	P-Value
6	rs13217619	28306671	3.62E-24
6	rs7746199	27261324	6.51E-23
6	rs71559070	28038929	2.15E-22
6	rs35848276	27521096	3.06E-22
6	rs17751184	27775028	5.04E-22
6	rs75782365	26408551	5.15E-22
6	rs57440165	26843517	2.44E-21
6	rs7749823	26158079	8.04E-21
6	rs13198474	25874423	5.52E-17
1	rs1782810	98502340	1.74E-15
10	rs7085104	104628873	2.68E-15
6	rs4712936	25417423	6.20E-15
10	rs34747231	104942244	1.14E-14

12	rs1024582	2402246	1.33E-13
15	rs7359276	78892661	1.95E-12
15	rs4702	91426560	4.64E-12
20	rs2103655	37425958	5.23E-12
18	rs72936314	53580677	2.28E-11
6	rs169738	33537546	2.88E-11
1	rs11210892	44100084	4.64E-11
2	rs11693094	185601420	6.24E-11
18	rs9636107	53200117	7.53E-11
11	rs55661361	124613957	1.12E-10
2	rs796364	200716119	1.19E-10
2	rs143911669	201068915	1.70E-10
7	rs12532143	111025948	2.01E-10
5	rs6868457	60550041	2.16E-10
3	rs75968099	36858583	2.40E-10
16	rs7185124	13750694	3.16E-10
11	rs75059851	133822569	3.67E-10
3	rs17620999	2561556	4.86E-10
14	rs12887734	104046834	5.10E-10
15	rs4586394	84822385	5.97E-10
15	rs11638554	85148231	9.01E-10
11	rs11027827	24375179	1.01E-09
22	rs8139773	39975691	1.31E-09
11	rs35274053	130718214	1.39E-09
11	rs7951870	46373311	1.43E-09
7	rs58120505	2029867	1.45E-09
11	rs7108770	46648432	1.62E-09
10	rs1163238	104943993	1.82E-09
2	rs6434928	198304577	1.92E-09
12	rs1615350	123650335	1.99E-09
5	rs16867576	88746331	2.00E-09
2	rs7599488	60718347	2.36E-09
12	rs679087	29917265	2.92E-09
18	rs11874716	52750688	3.04E-09
5	rs6879809	60826956	3.18E-09
3	rs832190	63842629	3.30E-09
3	rs940174	136309710	3.36E-09
8	rs13262595	143316970	4.68E-09
22	rs1058167	42538029	4.91E-09
4	rs13107325	103188709	5.03E-09
3	rs12488721	135992645	5.25E-09
18	rs1789589	53804156	5.52E-09
7	rs12704290	86427626	5.58E-09
11	rs12421382	109378071	5.73E-09
6	rs2213806	28457818	6.71E-09

14	rs10083370	104314182	6.86E-09
10	rs7893279	18745105	7.52E-09
12	rs2239063	2511831	1.25E-08
1	rs9428966	243667900	1.32E-08
3	rs34312605	180590297	1.42E-08
2	rs6704768	233592501	1.72E-08
5	rs7730110	153679191	1.78E-08
7	rs13233308	87244960	1.81E-08
2	rs11682175	57987593	1.93E-08
14	rs67981189	71472226	2.03E-08
6	rs10947428	33647058	2.09E-08
5	rs3849046	137851192	2.09E-08
5	rs3112532	152606570	2.49E-08
11	rs2514218	113392994	2.92E-08
14	rs2068012	30190316	3.13E-08
7	rs2192932	104653265	3.45E-08
3	rs2535627	52845105	3.70E-08
8	rs7833159	89587464	3.72E-08
6	rs12190758	93148341	3.92E-08
5	rs13361438	152007075	4.10E-08
1	rs2953329	244025999	4.11E-08
14	rs941521	99708876	4.16E-08
12	rs3741434	53605344	4.27E-08
7	rs6466055	104929064	4.36E-08
6	rs2022272	27103580	4.46E-08
2	rs9330316	110284236	4.59E-08
2	rs7577690	220045035	4.60E-08
4	rs7653924	170202862	4.67E-08
10	rs72841270	104642237	4.73E-08

Table S3. PRSice model fit at different thresholds of PRS-SCZ

PRS-SCZ					
Threshold	R2	P	Coefficient	Standard Error	Number of SNP
5.00E-08	0.0032082	0.420739	-25.98	32.2677	86
1.00E-07	0.00130388	0.610766	-18.5157	36.3781	97
1.00E-06	0.00011633	0.880556	-8.47224	56.3821	162
1.00E-05	0.00023525	0.831788	21.4583	101.023	332
0.0001	0.00040098	0.7807	-54.2767	194.954	858
0.001	0.00029035	0.812814	-97.9398	413.603	2518
0.01	0.00731406	0.23632	-1114.33	940.972	8315
0.05	0.0138329	0.10268	-2701.41	1655.28	20500
0.1	0.00513221	0.319934	-2143.52	2155.18	30334
0.5	0.00145097	0.597349	-2208.69	4181.41	70983
1	0.00107556	0.649305	-2368.41	5208.41	89305

PRSice linear regression models explaining phenotypic variance of SCZ with different PRS SCZ thresholds. The model fit (r2) increased by including more SNPs at the cost of a higher standard error. Comparing the GWAS hits threshold with lower significance thresholds revealed that the GWAS hits threshold provided a model fit better than all models until a significance threshold of p=0.01 and the second lowest standard error.

Figure S1. Correlations between PRS-SCZ and PRS-BIP at different cut-off thresholds in the final study sample (n=390)

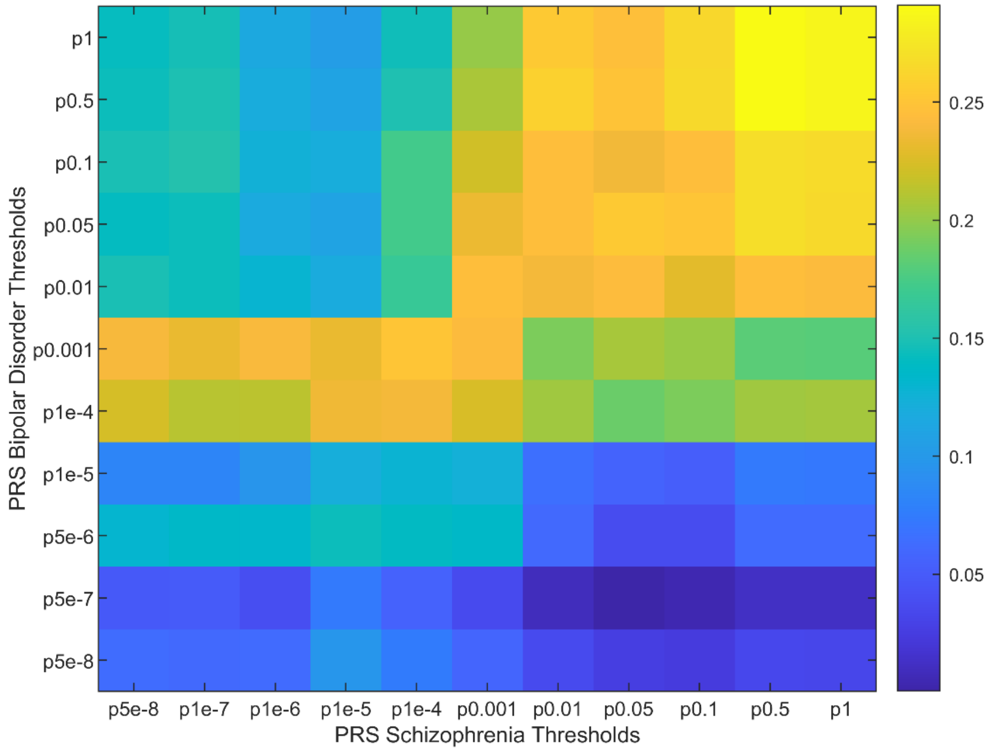

Model selection

The corrected Akaike information criterion (AIC) (7,8) and Bayesian information criterion (BIC) (9) were used to test whether models including quadratic age terms and interaction terms fit the data better than models with only lower degree age terms. The following three models were compared.

Model 1: including quadratic age terms and PRS-SCZ*age interaction terms (β_4PRS-SCZ*Age + β_5PRS-SCZ*Age^{2})

$$T_i = intercept + \beta_1 PRS\text{-}SCZ + \beta_2 Age + \beta_3 Age^2 + \beta_4 PRS\text{-}SCZ{*}Age + \beta_5 PRS\text{-}SCZ{*}Age^2 + \beta_6 Sex + \beta_7 PC10 + \beta_8 Scanner + \beta_9 BrainVolume + \varepsilon_i$$

Model 2: without quadratic age terms but including one PRS-SCZ*age interaction term (β_4PRS-SCZ*Age)

$$T_i = intercept + \beta_1 PRS\text{-}SCZ + \beta_2 Age + \beta_3 PRS\text{-}SCZ{*}Age + \beta_4 Sex + \beta_5 PC10 + \beta_6 Scanner + \beta_7 BrainVolume + \varepsilon_i$$

Model 3: including quadratic age terms but excluding PRS-SCZ*age interaction terms

$$T_i = intercept + \beta_1 PRS\text{-}SCZ + \beta_2 Age + \beta_3 Age^2 + \beta_4 Sex + \beta_5 PC10 + \beta_6 Scanner + \beta_7 BrainVolume + \varepsilon_i$$

Model	corrected AIC	BIC
Model 1 Age ^{2} + interaction terms	-909.25	1.0e+03 *-1267.1
Model 2 Age + interaction terms	-902.59	1.0e+03 *-1256.6
Model 3 Age^2 without interaction terms	-904.47	1.0e+03 *-1258.5

The corrected AIC and BIC values indicated that model 1 including quadratic age terms and PRS-SCZ*age, PRS-SCZ*age^2 showed the best model fit for the data.

Cellular composition of the cortex and PRS-SCZ effects on cortical thickness

We evaluated how the observed pattern of PRS-SCZ effects on cortical thickness relates to regional variations in the cellular compositions of the cortex. It is reported that regional variations in cortical thickness are not associated with the number of neurons (10). To further test this, we contrasted histological measurements of cortical thickness, neuronal density and neuropil (11). Cortical thickness and neuronal density were retrieved from table data on 39 cortical areas. Neuropil was estimated by scanning Von Economo photomicrographs and calculating the proportion of non-stained pixels within each cortical area. Subsequently, we focused on components of the neuropil, namely glial cell processes, axons, dendritic trees, neuron-to-neuron synapses i.e. in cortical tissue other than cell bodies or blood vessels.

Aggregation of PRS-SCZ effects on cortical thickness by cytoarchitectural type and functional network

We contextualized the PRS-SCZ effects on cortical thickness by cytoarchitectural types and intrinsic functional networks. Cytoarchitectural types were assigned to Von Economo areas (50,51), based on a recent re-analysis of Von Economo micrographs (35). Cortical types synopsize degree of granularity, from high laminar elaboration in koniocortical areas, six identifiable layers in eulaminate III-I, poorly differentiated layers in dysgranular and absent layers in agranular.

Functional networks were defined based on the Yeo atlas (36). The atlas reflects clustering of cortical vertices according to similarity in resting state functional connectivity profiles, acquired in 1000 healthy young adults. We assessed whether the PRS-SCZ effects on cortical thickness were stronger or weaker within each class, relative to spin permutations (52,53). Specifically, we calculated the median PRS-SCZ effect for each cytoarchitectural type and each functional network, then compared the median scores to a null model, which was constructed by randomly rotating the atlases across the cortical surface and re-evaluating the median scores (10,000 permutations). The medial wall was assigned as a NaN and not included in each permuted correlation (54). Statistical significance was deemed where $p_{spin}<0.025$ (two-tailed test).

Figure S2 and S3. Association between PRS-SCZ with surface area and cortical volume

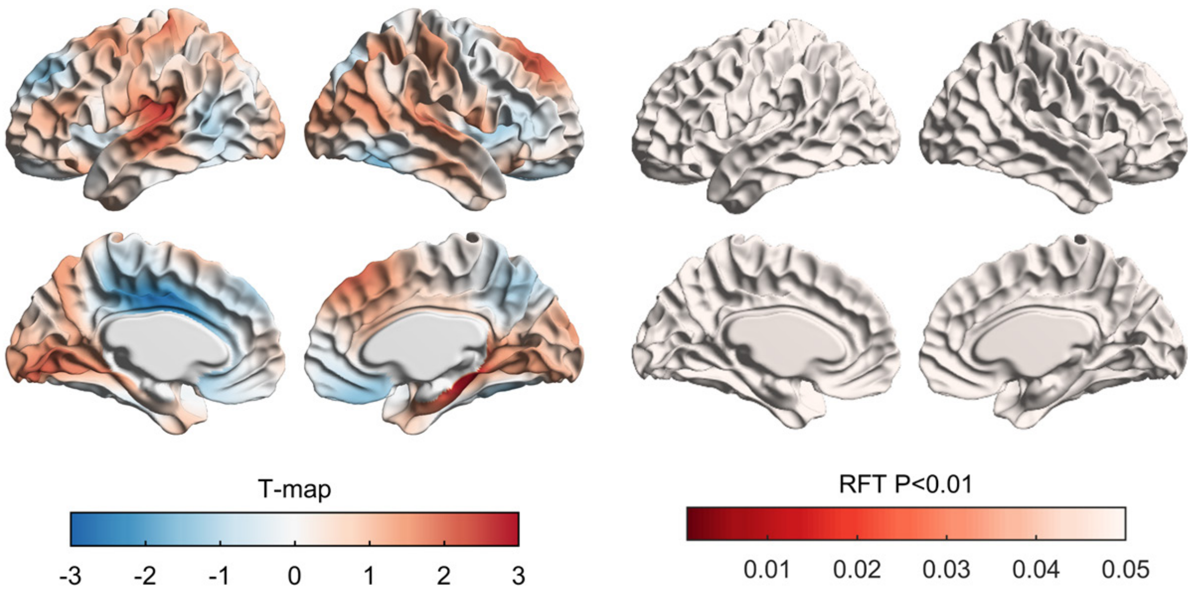

Supplementary Figure 2: C) Unthresholded (left) and cluster-level thresholded (right) maps show the association of PRS-SCZ with surface area using Random field theory (RFT) at cluster-level $p \leq .01$ (12,13).

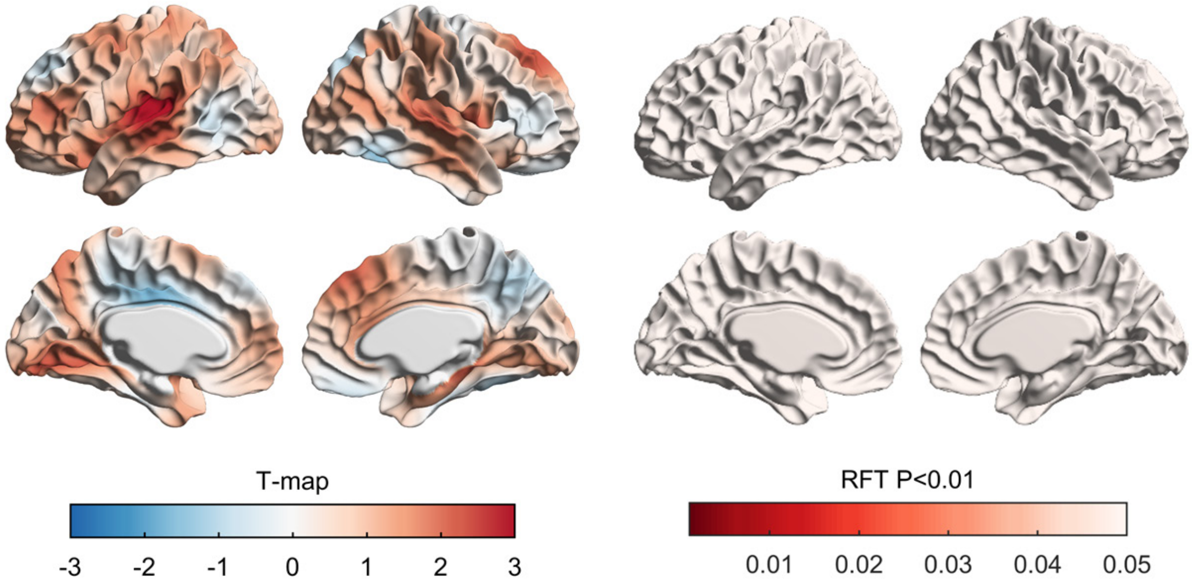

Supplementary Figure 3: Unthresholded (left) and cluster-level thresholded (right) maps show the association of PRS-SCZ with cortical volume using Random field theory (RFT) at cluster-level $p \leq .01$ (12,13).

.

Figure S4. Age-specific effects of PRS-SCZ on cortical thickness centering age in 1year bins.

Age 3 to 9

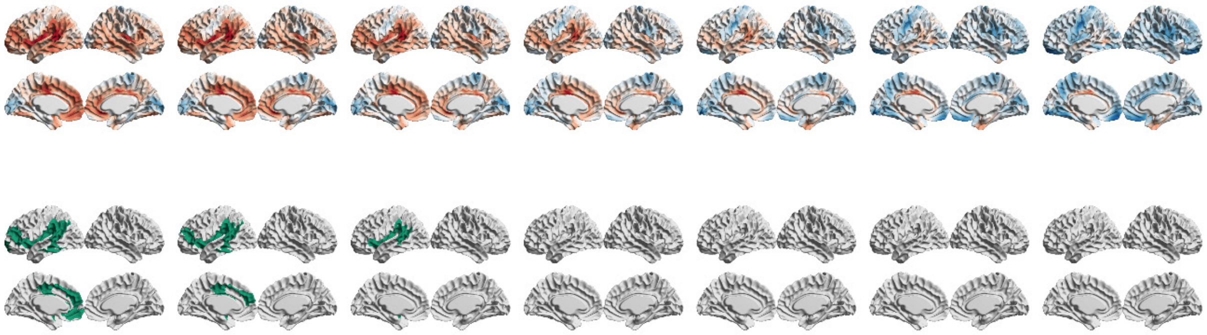

Age 10 to 16

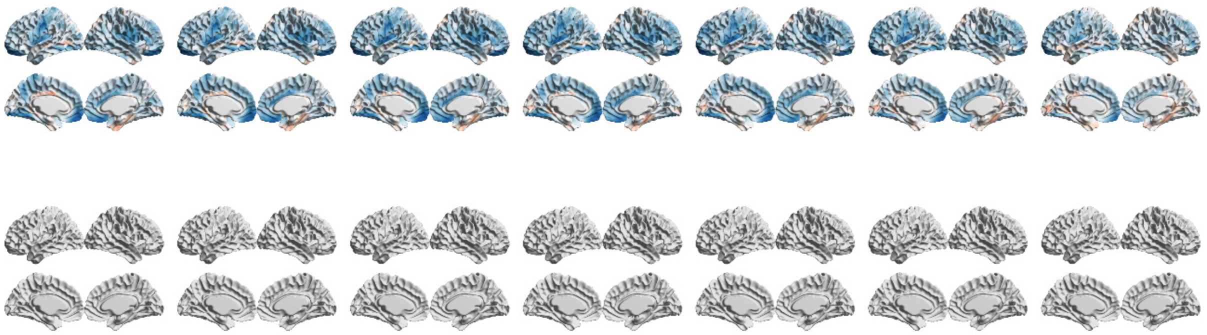

Age 17 to 21

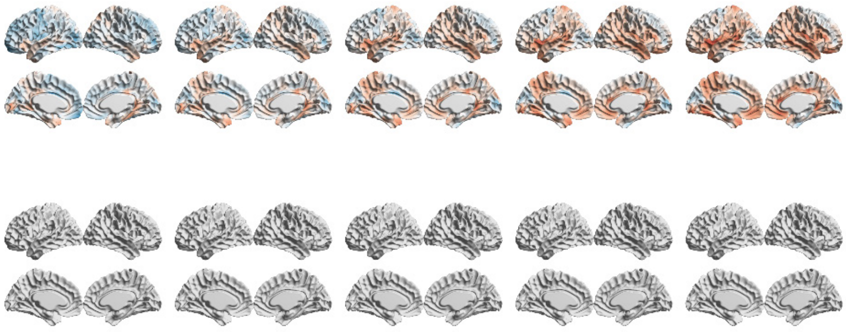

Supplementary Figure S4: Age-specific PRS-SCZ effect on cortical thickness Age 3 to 21 showing all 21 age bins. To analyze age-related changes in the PRS-SCZ effect on cortical thickness in the entire cohort (n=390), we repeated the main analysis (GLM (Eq.1) and iteratively shifted the age-centring from 3-21 in 1yr intervals (14). Upper rows unthresholed maps of PRS-SCZ effect on cortical thickness with different age-centering. Lower rows cluster-level thresholded maps of PRS-SCZ effect on cortical thickness with different age-centering using Random field theory at cluster-level $p \leq .01$.

Figure S5 Non-linear effects of PRS-SCZ across entire age range (3-21 yrs)

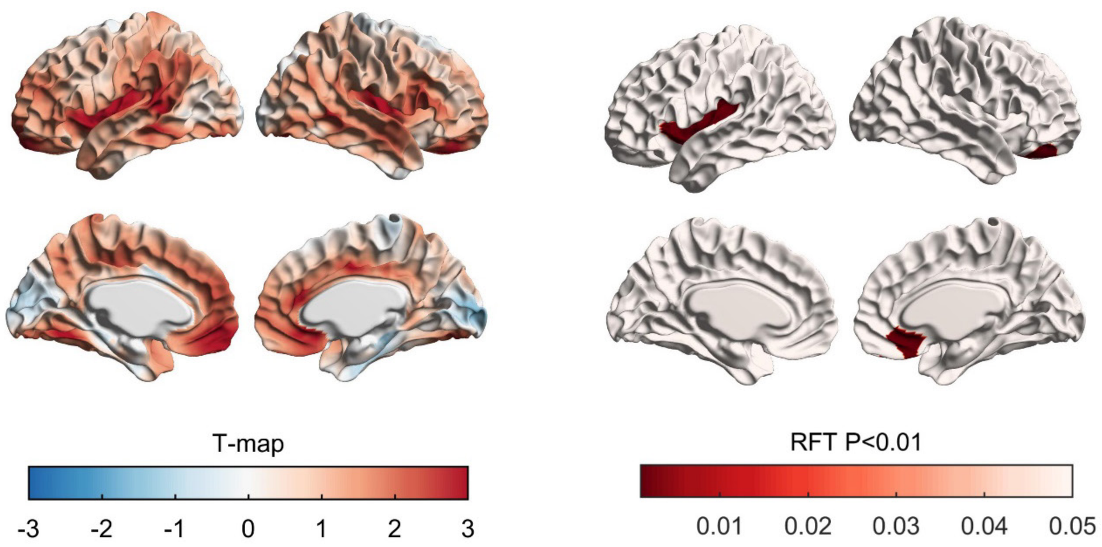

Supplementary Figure S5: Non-linear effects of PRS-SCZ across entire age range (3-21 yrs). Unthresholded (left) and cluster-level thresholded (right) maps show the association of PRS-SCZ-by-Age^2 with cortical thickness using Random field theory (RFT) at cluster-level $p \leq .01$ (12,13).

Supplementary References

1. Jernigan TL, Brown TT, Hagler DJ, Akshoomoff N, Bartsch H, Newman E, *et al.* (2016): The Pediatric Imaging, Neurocognition, and Genetics (PING) Data Repository. *NeuroImage* 124: 1149–1154.
2. Khundrakpam B, Vainik U, Gong J, Al-Sharif N, Bhutani N, Kiar G, *et al.* (2020): Neural correlates of polygenic risk score for autism spectrum disorders in general population. *Brain Commun* 2. https://doi.org/10.1093/braincomms/fcaa092
3. McCarthy S, Das S, Kretzschmar W, Delaneau O, Wood AR, Teumer A, *et al.* (2016): A reference panel of 64,976 haplotypes for genotype imputation. *Nat Genet* 48: 1279–1283.
4. Chang CC, Chow CC, Tellier LC, Vattikuti S, Purcell SM, Lee JJ (2015): Second-generation PLINK: rising to the challenge of larger and richer datasets. *Gigascience* 4: 7.
5. Ruderfer DM, Ripke S, McQuillin A, Boocock J, Stahl EA, Pavlides JMW, *et al.* (2018): Genomic Dissection of Bipolar Disorder and Schizophrenia, Including 28 Subphenotypes. *Cell* 173: 1705-1715.e16.
6. Choi SW, O'Reilly PF (2019): PRSice-2: Polygenic Risk Score software for biobank-scale data. *Gigascience* 8. https://doi.org/10.1093/gigascience/giz082
7. Akaike H (1969): Fitting autoregressive models for prediction. *Annals of the Institute of Statistical Mathematics* 21: 243–247.
8. HURVICH CM, TSAI C-L (1989): Regression and time series model selection in small samples. *Biometrika* 76: 297–307.
9. Schwarz G (1978): Estimating the Dimension of a Model. *The Annals of Statistics* 6: 461–464.

10. Herculano-Houzel S, Watson CR, Paxinos G (2013): Distribution of neurons in functional areas of the mouse cerebral cortex reveals quantitatively different cortical zones. *Front Neuroanat* 7. https://doi.org/10.3389/fnana.2013.00035

11. von Economo CF, Koskinas GN (1925): *Die Cytoarchitektonik Der Hirnrinde Des Erwachsenen Menschen*. J. Springer.

12. Hayasaka S, Phan KL, Liberzon I, Worsley KJ, Nichols TE (2004): Nonstationary cluster-size inference with random field and permutation methods. *Neuroimage* 22: 676–687.

13. Worsley KJ, Taylor JE, Tomaiuolo F, Lerch J (2004): Unified univariate and multivariate random field theory. *NeuroImage* 23: S189–S195.

14. Khundrakpam BS, Lewis JD, Kostopoulos P, Carbonell F, Evans AC (2017): Cortical Thickness Abnormalities in Autism Spectrum Disorders Through Late Childhood, Adolescence, and Adulthood: A Large-Scale MRI Study. *Cereb Cortex* 27: 1721–1731.
